# BuShen HuoXue Decoction Promotes Decidual Stromal Cell Proliferation via the PI3K/AKT Pathway in Unexplained Recurrent Spontaneous Abortion

**DOI:** 10.1155/2020/6868470

**Published:** 2020-10-05

**Authors:** Xiaoling Feng, Sha Jiang, WingTing Leung, Ling Wang, Hans Jürgen Gober, Lu Chen, Yang Zhang, Ling Wang

**Affiliations:** ^1^The First Affiliated Hospital of Heilongjiang University of Chinese Medicine, Harbin, China; ^2^The Heilongjiang University of Chinese Medicine, Harbin, China; ^3^Hospital and Institute of Obstetrics and Gynecology, Fudan University, Shanghai, China; ^4^The Academy of Integrative Medicine, Fudan University, Shanghai, China; ^5^Shanghai Key Laboratory of Female Reproductive Endocrine-related Diseases, Shanghai, China; ^6^The Eighth Clinical Medical College of Beijing University of Chinese Medicine, Xiamen, China; ^7^Department of Pharmacy, Neuromed Campus, Kepler University Hospital, Linz 4020, Austria

## Abstract

BuShen HuoXue decoction (BSHXD) has been used to treat patients with unexplained recurrent spontaneous abortion (URSA). However, the chemical compounds and mechanism by which BSHXD exerts its therapeutic and systemic effects to promote the proliferation of decidual stromal cells (DSCs) has not been elucidated. This work sought to elucidate the cellular and molecular mechanism of BSHXD in terms of inflammatory factors IL-17A in DSCs *in vitro* because of the critical roles of inflammation, apoptosis, and immunity in the development and progression of pregnancy loss. Twelve migratory chemical compounds from BSHXD extract were qualitatively analyzed by high-performance liquid chromatography (HPLC). DSCs were collected from normal early pregnancy (NEP) and URSA to determine whether BSHXD affects IL-17A/IL17RA via the PI3K/AKT pathway. Abnormal apoptosis and activated p-AKT were observed in URSA DSCs. RhIL-17 A, LY294002 (a PI3K pathway inhibitor), and BSHXD were individually or simultaneously administered in NEP DSCs, suggesting that BSHXD restored cell proliferation without excessive stimulation and IL-17A promotes proliferation via the PI3K/AKT pathway. Using the same intervention in URSA DSCs, qRT-PCR measured the upregulated mRNA levels of IL-17 A/IL-17RA, PI3K, AKT, p-AKT, PTEN, Bcl-2, and Bcl-xL and downregulated mRNA levels of BAD and ACT1 after treatment with BSHXD. We demonstrated that BSHXD affected IL-17A/IL-17R via PI3K/AKT pathway to promote the proliferative activity of DSCs in URSA. These results provide a new insight to further clarify the relationship between inflammation and apoptosis and the mechanism of imbalance in the dynamic equilibrium between Th17/Treg immune cells at the maternal-fetal interface.

## 1. Introduction

Recurrent spontaneous abortion (RSA) refers to consecutive abortions occurring before 20 weeks with the same biological father [1]. RSA is increasingly associated with a variety of mechanisms, such as genetic abnormalities, endocrine disorders, infectious inflammation, anatomical malformation, thrombophilic tendency, and environmental factors [2]. Up to 50% of RSA cases occur in the absence of common obstetrical and gynecological complications [3] and are therefore referred to as unexplained RSA (URSA). Treatment strategies such as aspirin and low-molecular-weight heparin (LMWH) have recently been recommended by practical guidelines for URSA and had provided some curative effects [4]. Targeted drugs such as TNF-*α* inhibitors and granulocyte colony-stimulating factor (G-CSF) are potential drugs for URSA [5]. In addition to the many advances in URSA treatment in Western medicine, increasing attention is being given to traditional Chinese medicine (TCM) because of its advantages in enhancing fertility and live birth rates. TCM is currently recognized as a prevalent complementary and alternative treatment for URSA in the Western world [6].

TCM is the mainstream of healthcare throughout East Asia, and complex herbal formulations have been applied to treat diseases for 5,000 years. Compared with Western medicine, TCM has a unique theory for the diagnosis and treatment of repeated pregnancy loss. A systematic review on TCM and pregnancy loss suggested that Chinese herbal medicine can effectively enhance estrogen and progestin levels [7, 8], improve endometrial receptivity [9, 10], and ultimately influence pregnancy outcomes [11, 12]. BuShen HuoXue decoction (BSHXD) is an efficacious prescription to prevent miscarriage in URSA and assist reproduction [13]. Previous studies of its mechanism in rats have provided a theoretical and practical reference for the comprehensive roles of BSHXD in angiogenesis, hormone management, endometrial receptivity, and reproductive outcomes [14].

Pregnancy includes an ongoing balance between immune and inflammatory responses [15]. In the current study, we found that decidual stromal cells (DSCs) represent the majority (approximately 70%) of decidual cells. In addition, these DSCs form the decidua surface [16] and exert multiple effects during pregnancy [17]. The balance between DSC apoptosis and proliferation plays a vital role in pregnancy maintenance, and these DSCs are converted to decidual cells in preparation for embryo implantation and development [18]. IL-17 A is crucial in inflammation, autoimmune diseases, and reproduction [19] and was recently confirmed to be associated with *γδ*T cells [20] and recruited by DSCs [21]. In addition, DSCs were reported to promote trophoblast proliferation and IL-17 secretion in women with normal pregnancies [22]. Moreover, DSCs exhibit migration and chemotaxis involving the PI3K/AKT pathway [23, 24].

In our previous clinical studies, we observed differences in IL-17A levels in the peripheral sera of NEP and URSA patients and the abnormal apoptosis of DSCs from URSA patients [25]. Abnormal upregulation of IL-17A and IL-17R in the peripheral blood and abnormal apoptosis of DSCs in URSA patients [26] was attenuated by BSHXD therapy. Therefore, we hypothesize that BSHXD acts on IL-17A to ameliorate abnormal apoptosis by regulating the PI3K/AKT pathway. To further reveal the effects and underlying genetic pathway of BSHXD in humans, we treated DSCs with serum containing BuShen HuoXue decoction (BSHXDS) *in vitro* and assessed the consequences of BSHXDS treatment on the proliferation of DSCs, levels of the cytokines IL-17 A/IL-17R, and changes in PI3K/AKT signal transduction.

## 2. Materials and Methods

### 2.1. BSHXDS Preparation

Sprague-Dawley (SD) rats were obtained from the Animal Laboratory of Heilongjiang University of Chinese Medicine. Thirty adult female rats were raised in a specific-pathogen-free environment with a 12 h light: dark cycle for one week. All procedures involving rats were approved by the animal ethics committee of the Heilongjiang University of Chinese Medicine. The composition of BSHXD is listed in Table 1. These compounds were obtained from the First Affiliated Hospital of Heilongjiang University of Chinese Medicine (Harbin, China). BSHXD was decocted with boiling water twice at 100°C for 30 min and sufficiently mixed with donkey-hide glue/gelatin. The mixed-decoction was centrifuged at 12,000 r/min for 10 min. The remaining serum supernatant was added to a bottle for analysis. The same method was used to extract the individual components.

Seven SD rats were divided into two groups, the BSHXDS group (*N* = 6) and the control serum group (*N* = 1), and received 0.8 g of BSHXD/100g of body weight or an equal amount of physiological saline, respectively. These treatments were administered daily by oral gavage for three days, and hepatic portal venous blood was collected on the third day during the last administration of BSHXD for 30 min. After centrifugation, collection, filtration, and inactivation, the remaining serum supernatant was stored at −80°C.

### 2.2. HPLC Analysis of Identified Compounds

High-performance liquid chromatography (HPLC) was used to identify the active biomolecules of BSHXDS. First, the chromatographic peaks of BSHXD and its monomeric components were compared to select ion peaks. Second, the ion peaks that were present in the supernatant serum of BSHXD-treated rats but not in the serum of rats that received normal saline were selected. These peaks were further compared with the ion peaks of BSHXD *in vitro* to confirm the effective components of BSHXDS in blood and to calculate the time points corresponding to the most effective components. Enkephalin contrast solution leucine-enkephalin (concentration 1 ng/ml) was applied at 20 *μ*l/min. Detailed conditions are described in Table 2.

### 2.3. Study of Tissue Samples and Cell Culture

Clinical samples from patients aged 18 to 30 years were collected from The First Affiliated Hospital of Heilongjiang University of Chinese Medicine in China between June 2017 and Feb 2018. Tissues were collected from 10 cases of normal early pregnancy (NEP) (women who have a history of pregnancy and childbirth and no history of spontaneous abortion) and 10 cases of unexplained recurrent spontaneous abortion (URSA) between eight and ten weeks of gestation. None of the embryos had chromosomal abnormalities. None of the patients had taken any medicine. All sample collection and experimental setup protocols were permitted by the Ethics Committee of the First Affiliated Hospital of Heilongjiang University of Chinese Medicine.

DSCs isolated from the tissues were cultured *in vitro* as follows. Decidual tissues were washed and fragmented in PBS (HyClone, USA). Collagenase IV and DNase І (1 : 25) were used to digest the samples for 30 min at 37°C in a water bath with agitation (40–60 rpm/min). After centrifugation at 1,200 r/min for 10 min, the cells were suspended in DMEM/F12 (HyClone, USA) (containing 10% FBS (Gibco-BRL, USA) and 1% Tri-antibody) medium. The medium was covered with Percoll and centrifuged at 2,000 r/min for 20 min. The cells were washed and resuspended in DMEM/F12 and maintained in 5% CO_2_ atmosphere at 37°C. The adherent cells after overnight culture were purified. The medium was replenished every two to three days.

### 2.4. Apoptosis and CCK-8 Assay

DSCs were washed with PBS, digested with trypsin at 37°C for 1 min, and centrifuged (1,500 r/min) for 7 min. After resuspension, the cells were counted and centrifuged. One *μ*l of PE (BD Bioscience, China) and 2.5 *μ*ml of 7-ADD were added, except for the control cells, and cells were incubated away from light for 15 min. Diluted binding buffer (400 *μ*l) was added to each tube before analysis. All steps were completed in 30 min and repeated three times. The apoptosis rate was determined by flow cytometry.

DSCs were incubated in a 96-well plate (1×104 cells/well) and cultured for 72 h after the addition of DMEM/F12 medium. CCK-8 reagent (Weiao Biotechnology, China) was added to the different groups at various concentrations. The reagent was mixed with DMEM/F12 at a ratio of 1 : 9, and then 100 *μ*l of the solution was quickly added to each well after mixing. The plate was incubated at 37°C for 1–4 h, and the OD value of each well was detected at 450 nm.

### 2.5. Experimental Scheme and Drug Administration

For the subsequent drug intervention, recombinant human IL-17A (Peprotech, USA) cytokines were dissolved in PBS containing 5% trehalose for storage, and the experimental concentration gradients were set at 0, 10, 20, 30, 40, and 50 *μ*g/ml. The PI3K pathway inhibitor LY294002 (Merck Millipore, Germany) was prepared at a storage concentration of 10 mM, and the experimental concentration gradients were 0, 1, 10, 25, and 50 *μ*M. The control group of cells received normal saline. Different concentrations of RhIL-17A, LY294002, and BSHXD were individually or simultaneously administered in NEP or URSA DSCs.

The concentration gradient of the drug intervention group was as follows: 10% control, 7.5% control + 2.5% BSHXDS, 5% control + 5% BSHXDS, 2.5% control + 7.5% BSHXDS, and 10% BSHXDS. All of the URSA samples were incubated for 72 h, and the inhibitors were added to the cells at 1 h before rhIL-17A.

### 2.6. RNA Isolation and qRT-PCR Analysis

Total RNA was isolated from DSCs in a 96-well plate (5 × 10^6^ cells/well). After treatment with BSHXDS for 72 h. RNAiso Plus (TaKaRa Biotechnology, Japan) was used to extract RNA from the cell medium according to the manufacturer's recommendation. A nucleic acid/protein analyzer was used to measure the concentration and purity of the RNA. cDNA synthesis was performed with PrimeScript RT Master mix (TaKaRa Biotechnology, Japan) in a reaction volume of 10* μ*l when a suitable RNA concentration and purity were achieved. SYBR Premix Ex Taq (TaKaRa Biotechnology, Japan) was performed for quantitative real-time PCR (qRT-PCR) analysis following the manufacturer's instructions. The reverse transcription reaction conditions were as follows: 30 s at 95°C, 40 cycles of 5 s at 95°C, and 30 s at 60°C. The sequences of primer pairs are shown in Table 3.

### 2.7. Western Blotting

DSCs were cultured in a 6-well plate (1 × 10^6^ cells/well). Protein was extracted from NEP and URSA DSCs. The protein level of PI3K and p-AKT was measured. Then, rhIL-17A was added at 10, 20, 30, 40, 50, or 60 min before protein extraction. Protein extraction scheme was followed. Cell lysates were prepared and quantified. Equivalent amounts of protein samples were separated by 10% sodium dodecyl sulfate-polyacrylamide gel electrophoresis (SDS-PAGE) (EpiZyme Scientific, USA). The proteins were transferred onto PVDF membranes. PVDF membranes were blocked with 5% nonfat dry milk at room temperature, and anti-GAPDH, anti-PI3K, anti-AKT1, and anti-p-AKT (Cell Signaling Technology, USA) antibodies were added to primary antibody dilution buffer and incubated at 4°C overnight. The membranes were then washed with TBST three times for 5 min each. Secondary antibody (Bioworld Technology, China) was added to dilution buffer and incubated for 1 h, and the membrane was washed with TBST three times for 15 min before visualization. Anti-GAPDH was used as the control.

### 2.8. Statistical Analysis

All experimental results are presented as the mean ± standard error of the mean and were analyzed by SPSS 21.0 software (SPSS Inc. Chicago, IL, USA). Different groups were compared by one-way analysis of variance (ANOVA). The independent-sample *t*-test was used for comparisons between groups. A *p* value < 0.05 was considered statistically significant.

## 3. Results

### 3.1. Identification of Twelve Compounds in BSHXDS by HPLC Analysis

**Figure 1:**
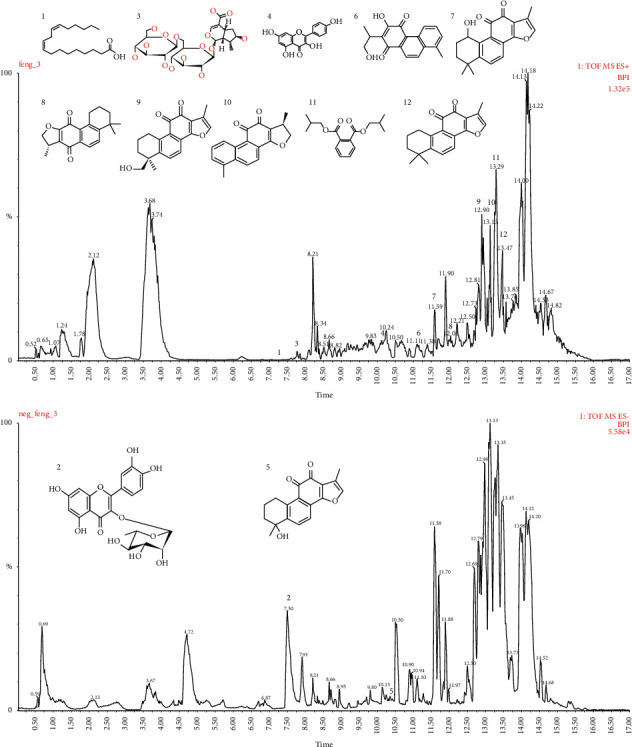
The HPLC fingerprints of BSHXDS components: (1) linoleic acid; (2) quercetin-3-O-*α*-L-rhamnopyranoside; (3) loganic acid 6′-O-*β*-D-glucoside; (4) kaempferol; (5) tanshinol B; (6) 2-(1-methyl-2-hydroxyethyl)-3-hydroxy-8-methylphenanthrene-1,4-dione; (7) hydroxytanshinone II A; (8) isotanshinone III; (9) tanshinone II B; (10) 15,16-dihydrotanshinone I; (11) diisobutyl phthalate; (12) tanshinone II A.

To compare the different chromatographic peaks of BSHXD, Chinese herbal medicine monomers, and BSHXDS, we collected BSHXDS at 30 min and identified 12 compounds from 75 (Figure 1). Twelve drug prototype compounds were identified in the blood, as shown in Table 4.

### 3.2. Abnormal Apoptosis of DSCs in URSA

**Figure 2:**
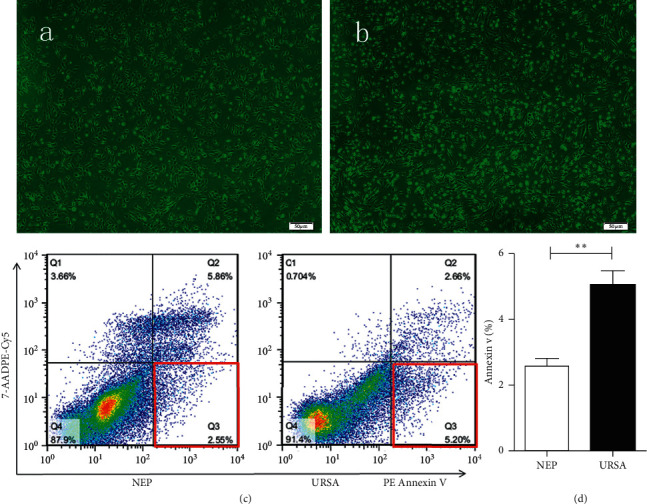
The morphology of DSCs in NEP (a) and URSA (b) under electron microscope. Flow cytometry showed a PE antibody-positive region in Q3 at the early stage (c). The apoptosis rate of DSCs in URSA (5.20%) and NEP (2.55%) (d). ^*∗∗*^*p* < 0.01.

The morphology of DSCs was observed by electron microscopy, as shown in Figure 2(a) and 2(b), revealing more irregularly spindle-shaped, poorly shaped, and unsaturated cells and cell fragments in the DSCs of URSA compared with NEP-derived DSCs. Flow cytometric analysis was used to calculate the apoptosis rate of DSCs at the early stage of apoptosis as shown in Figures 2(c) and 2(d). The apoptosis rate of DSCs in URSA (5.20%) was higher than that of DSCs in NEP (2.55%) at the early stage (*p* < 0.01) (Figure 2(c) Q3).

There were still a few apoptotic cells at the early stage of apoptosis in NEP DSCs (Figure 2(a)), and the NEP DSCs had an even higher apoptotic ratio (5.86%) than URSA DSCs (2.66%) (Figure 2(c), Q2). It is possible that some of the DSCs extracted from the decidual tissue after curettage had died. Moreover, an increased percentage of viable cells in the same culture bottle and the same electron microscope field indicated faster apoptosis.

### 3.3. rhIL-17A and LY294002 Regulate Proliferation in DSCs via PI3K/AKT Signaling

**Figure 3:**
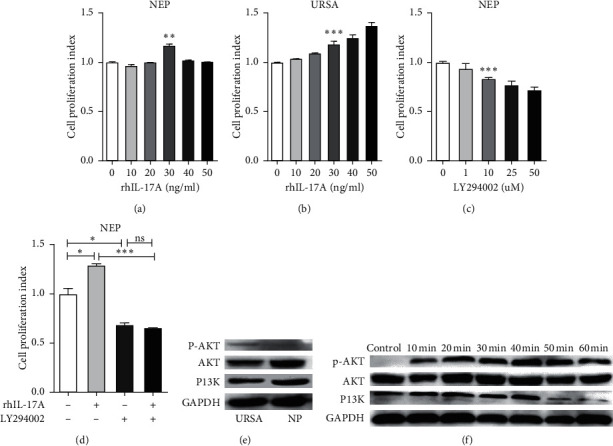
Cell proliferation index of NEP (a) or URSA (b) with different concentrations of rhIL-17A, LY294002 (c), and both (d). ^*∗*^*p* < 0.05; ^*∗∗*^*p* < 0.01; ^*∗∗∗*^*p* < 0.001; NS, no significant difference (*p* > 0.05). Representative immunoblots in PI3K/AKT signaling and average expression of PI3K and p-AKT in NEP and URSA DSCs (e) and treated with rhIL-17A (30 ng/ml) for 1 h in NEP DSCs (f).

In this study, we hypothesized that IL-17A ameliorates abnormal apoptosis by regulating PI3K/AKT pathway. The cell proliferation index of DSCs in NEP and URSA induced by rhIL17-A is shown in Figure 3(a) and 3(b), among which the percentage of 30 ng/ml rhIL17-A was significantly different in cell proliferative index compared to the control (*p* < 0.01) (*p* < 0.001). To determine whether PI3K/AKT signaling could affect the proliferation of DSCs *in vitro*, the cell proliferative index of DSCs in NEP induced by LY294002 (a PI3K pathway inhibitor) was subsequently measured. As shown in Figure 3(c), treatment with 10 *μ*M LY294002 resulted in the lowest cell proliferation index (*p* < 0.001). In addition, we detected the activated p-Akt in URSA DSCs (Figure 3(e)).

We then stimulated NEP DSCs with rhIL-17A (30 ng/ml), and western blot analysis indicated that rh-IL-17A could activate the PI3K/AKT signaling pathway (Figure 3(f)). Compared to that in the control group, rhIL-17A significantly promoted the proliferation index of NEP DSCs (*p* < 0.05) (Figure 3(a)) and URSA (*p* < 0.001) (Figure 3(b)), whereas LY294002 significantly decreased the cell proliferation index of NEP DSCs (*p* < 0.001) (Figure 3(c)). However, the suppressive influence of LY294002 could not be reversed by rhIL-17A (*p* > 0.05) (Figure 3(d)).

### 3.4. BSHXDS Promotes the Proliferation of DSCs in URSA

**Figure 4:**
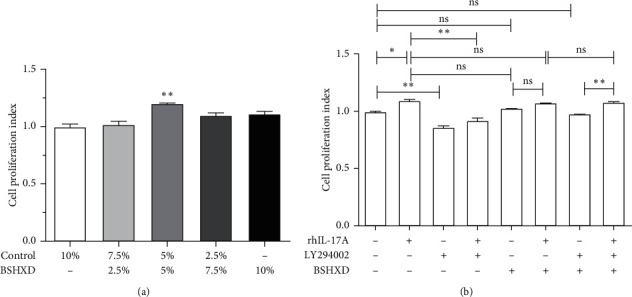
(a) Average cell proliferation indexes of DSCs in URSA treated with different concentrations of BSHXD and control. (b) Proliferation indexes of DSCs treated with or without rhIL-17A (+) compared with rh-17A (+), LY294002, and BSHXD (+) is ns. ^*∗*^*p* < 0.05; ^*∗∗*^*p* < 0.01; ns, *p* > 0.05.

Since rhIL-17A regulates in DSC proliferation via PI3K/AKT signaling, we conducted CCK-8 cell proliferation assays to assess the effect of BSHXDS on the proliferation of DSCs in URSA. IL-17A expression and PI3K/AKT signaling were altered following treatment with BSHXDS at various concentrations for 72 h, as described previously. As shown in Figure 4, 5% control +5% BSHXDS treatment obviously restored the proliferative activity of DSCs in URSA (Figure 4(a)) (*p* < 0.01). This effect did not excessively stimulate the proliferation of DSCs after the addition of rhIL-17A (Figure 4(b)) (*p* > 0.05). No obvious difference in proliferation was found between DSCs treated with BSHXD, LY294002 + BSHXD, compared with control DSCs, confirming that BSHXDS eliminated the effects of inhibitors and reactivated PI3K/AKT signaling (*p* > 0.05). Furthermore, no significant difference was also found between DSCs treat with BSHXD, rh-IL-17A + BSHXD, compared to those treated with rh-IL-17A, suggesting that BSHXD restored cell proliferation without excessive stimulation (*p* > 0.05). A difference in the proliferation of DSCs in the LY294002 + BSHXDS and LY294002 + BSHXDS + rhIL-17A groups was also observed, indicating that BSHXDS eliminated the effect of LY294002 and recovered the effect of IL-17A in promoting the proliferation of DSCs (Figure 4(b)).

### 3.5. BSHXDS Effects on IL-17A/IL-17RA via PI3K/AKT Signal Transduction

**Figure 5:**
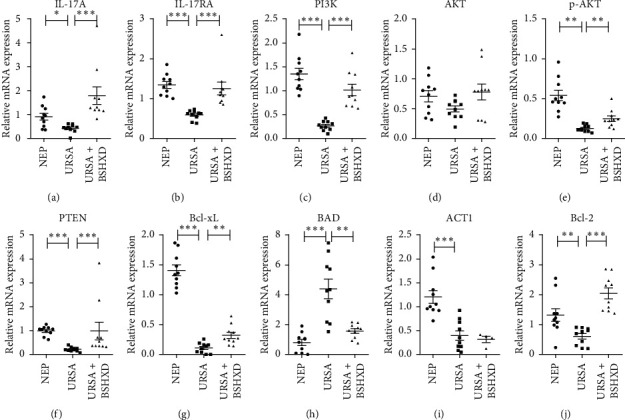
Real-time PCR was performed to assess the relative expression of IL-17A (a), IL-17RA (b), PI3K (c), AKT (d), p-AKT (e), PTEN (f), Bcl-xl (g), BAD (h), ACT1 (i), and Bcl-2 (j) after treatment with BSHXD for 72 h (^*∗*^*p* < 0.05, ^*∗∗*^*p* < 0.01, and ^*∗∗∗*^*p* < 0.001).

All the previously mentioned results show the potential role of IL-17A in regulating PI3K/AKT signal transduction. Quantitative real-time fluorescence PCR showed that the downregulated expression levels of IL-17A/IL-17RA in URSA DSCs were upregulated to varying degrees after BSHXDS treatment for 72 h (Figure 5(a) and 5(b)). We therefore investigated changes in the expression of the molecules PI3K, AKT, p-AKT, PTEN, Bcl-xL, ACT1, Bcl-2, and BAD in the PI3K/AKT pathway.

The downregulated levels of cytokines in the PI3K/AKT pathway (PI3K, AKT, p-AKT, PTEN, Bcl-xL, ACT1, and Bcl-2) were increased after BSHXDS administration (*p* < 0.01, *p* < 0.001) (Figures 5(c)–5(g) and 5(j)). The expression level of the antiapoptotic molecule Bcl-xL was increased when this pathway was activated, while the expression level of the proapoptotic molecule BAD was decreased (*p* < 0.01, *p* < 0.001) (Figure 5(g)). We concluded that these cytokines helped regulate the PI3K/AKT pathway to restore the proliferative ability of DSCs.

## 4. Discussion

URSA occurs in 5% of couples, and the failure of a clinically detectable pregnancy usually occurs within the first 20 weeks after conception. Due to its indeterminate etiology and the lack of evidence-based diagnostic and systematic treatment strategies, URSA is a growing reproductive disorder that seriously endangers the physical and mental health of patients and challenges clinicians. The BuShen HuoXue method was first described by Professor Zhang Daning, which originated from Huangdi's Internal Classic, was developed by Zhang Zhongjing, and finally refined by Zhang Jingyue and Wang Qingren. Kidney essence, qi, and blood in the reproductive system provide substance, direction, and energy. Drugs in herbal medicines reinforce the kidney and activate blood circulation, which are similar to endocrine hormones. They not only play a direct role in ovarian function but also regulate immunomodulatory function. Furthermore, these drugs promote follicular development, ovulation, and luteal formation and growth, supplement estrogen and progesterone [27], and improve uterine receptivity [28].

Apoptosis is a form of programmed cell death required to remove infected, damaged, and unwanted cells, to assist cells in maintaining a balance between development and aging and to regulate the number of cells in tissues [29,30]. The BCL-2 family of proteins includes survival-promoting region restrictors (Bcl-2, Bcl-xL) and upstream markers of BH3 in individual apoptosis-promoting regions (Bid, Bim, BAD, etc.). These proteins regulate cell survival and proliferation in the mitochondrial apoptosis pathway [31]. PI3K/AKT signal transduction is a classic mitochondrial apoptosis pathway involved in cell survival and proliferation regulation. It not only regulates apoptosis by inhibiting FKHR, NF-*κ*B, YAP, and BAD [32] but also contributes to AKT/PKB signal transduction by activating p-AKT [33,34]. AKT also regulates cell survival by directly inhibiting proapoptotic signals, such as BAD [35], and by phosphorylating PIP2, producing PIP3. PIP3, which is located in the membrane, activates AKT to produce p-AKT. However, PTEN can antagonize p-AKT by dephosphorylating PIP3, producing PIP2 [36]. LY294002 significantly inhibits the activity of PI3K and thus decreases the activity of PIP3 and p-AKT [37]. In healthy cells, p-AKT inhibits apoptotic signals and the proapoptotic protein BAD [38]. IL-17A, a proinflammatory factor that can induce an abnormal respiratory neutrophil response to antigen stimulation [39], is associated with a variety of inflammatory states, such as those in autoreactive diseases [40], metabolic abnormalities, and cancer [41]. Pathological products of IL-17A can lead to excessive inflammation and significant tissue damage [42]. Animal experiments in a typical model of URSA (CBA/J ×∗DBA/2 mice) revealed that IL-17 injection into the middle abdomens of normal pregnant mice increased the abortion rate, whereas injection of IL-17-neutralizing antibody decreased the abortion rate. Obviously, circulating IL-17 can induce abortion to some extent [43]. Under the stimulation of IL-17, ACT1 was recruited to IL-17R through the interaction of SEFIR-SEFIR region to activated pathway.

In NEP DSCs, rhIL-17A stimulated the proliferation of DSCs. First, we treated NEP DSCs with LY294002 and observed that cell proliferation was attenuated. The inhibitory effects of LY294002 on proliferation were not restored when rhIL-17A was added, indicating that IL-17A functions through the PI3K/AKT pathway. Next, we detected the protein levels of PI3K and p-AKT after stimulation with rhIL-17A by western blotting, which further demonstrated that the PI3K/AKT signaling pathway plays a role in DSC proliferation. Finally, treatment of NEP DSCs with BSHXD did not affect proliferation, confirming the safety of BSHXD. In addition, abnormal downregulated level of IL-17 A/IL-17RA and the activated p-Akt protein in URSA DSCs was observed. Therefore, We detected IL-17A, ACT1, and IL-17RA and PI3K/AKT related factors to ascertain the effects of BSHXD via IL-17 A/IL-17RA on PI3K/AKT pathway in URSA DSCS. As shown in Figure 4, BSHXD eliminated the inhibitory effects of LY294002 and restored IL-17A-mediated activation in URSA DSCs. To further determine whether BSHXD and IL-17A have a synergistic influence on the proliferation of DSCs via the PI3K/AKT pathway, we performed real-time PCR to determine the mRNA levels of IL-17 A/IL-17RA, ACT1, PI3K, AKT/p-AKT, PTEN, Bcl-xL, Bcl-2, and BAD.

The mRNA expression levels of PI3K, p-AKT, and PTEN, members of PI3K/AKT pathway, were lower in URSA DSCs than in NEP DSCs. A downward trend in the expression of AKT was observed, but the difference in AKT expression did not reach significance. BSHXD upregulated the expression of PI3K, p-AKT, and PTEN. Interestingly, PTEN, an inhibitor of p-AKT, should have been expressed at low levels, but we found that BSHXD upregulated PTEN to the same level in URSA DSCs and NEP DSCs. These results indicate that proliferation requires bidirectional regulation because excessive proliferation, such as that due to tumor-like changes, should be as harmful to pregnancy as insufficient proliferation. Bcl-xL and BAD, which regulate apoptosis, are located downstream of the PI3K/AKT signaling pathway. Activation of the PI3K/AKT signaling pathway can promote Bcl-xL expression and inhibit BAD activation. Abnormally elevated BAD and downregulated Bcl-xL levels were observed in URSA DSCs, and BSHXD was shown to regulate both PI3K/AKT and Bcl-xL. Low BAD levels promoted DSC proliferation and inhibited apoptosis. Downregulation of the mRNA levels of BAD and upregulation of the mRNA levels of IL-17 A/IL-17RA, PI3K, AKT/p-AKT, PTEN, and Bcl-xl confirmed the synergistic effects of BSHXD and IL-17A on DSC proliferation. We therefore concluded that BSHXD may play a therapeutic role in URSA by upregulating the expression of IL-17RA and activating the PI3K/AKT pathway in DSCs.

Although our present study successfully identified twelve chemical compounds in BSHXD and confirmed their systemic effects, few studies of the critical effects of these compounds have been performed. Notably, IL-17A at a 30 ng/ml concentration had immunomodulatory effects on the maternal-fetal interface, but we did not confirm these effects. We can refer only to previous reports, which showed that IL-17 (20 ng/ml) increases the secretion of IL-6 and IL-8, inducing rheumatoid arthritis (RA) [44], and has immunomodulatory effects on multiple sclerosis (MS) [45]. In addition, whether BSHXD affects IL-17A/IL-17RA via PI3K/AKT signaling in diverse decidual immune cells (DICs), especially *γδ* T cells, to maintain pregnancy and the potential mechanism of this effect remains unknown. Through further experiments, we will explore the most active compounds in BSHXD, the compatibility of BSHXD components, and the interaction of BSHXD with DICs. However, *in vitro* research is still needed to support the application of BSHXDS as a possible remedy for URSA.

## 5. Conclusion

In conclusion, this work identified twelve compounds in BSHXD by chromatographic analysis and demonstrated that administration of BSHXD restored cell proliferation without excessive stimulation and had an effect on IL-17 A/IL-17RA via the PI3K/AKT signaling pathway. In brief, BSHXDS inhibits the excessive secretion of IL-17A and inflammatory effects to regulate immunity at maternal–fetal interface, activate the PI3K/AKT pathway in DSCs, promote the proliferation of DSCs, and ultimately prevent abortion.

## Figures and Tables

**Table 1:** The composition of BSHXD.

Crude herb	Scientific name	Content (g)
Radix salviae miltiorrhizae	*Salvia miltiorrhiza Bunge*	15
Chinese Dodder Seed	*Cuscuta chinensis Lam*	15
Himalayan Teasel Root	*Dipsacus asperoides C. Y. Cheng et T. M. Ai*	15
Herba taxilli	*Taxillus chinensis* (DC.)*Danser*	15
Radix Astragali	*Astragalus membranaceus (Fisch.) Bge.*	15
Donkey-hide Glue	*Equus asinus Linnaeus*	10

**Table 2:** HPLC conditions.

Time	Speed	*A*%	*B*%
0	0.4	2	98
6	0.4	8	92
9	0.4	35	65
10	0.4	40	60
12	0.4	70	30
15	0.4	100	0

**Table 3:** Primer pairs.

Gene	Prime sequence (5′-3′)
GAPDH forward primer	ACAGTCAGCCGCATCTTC
GAPDH reverse primer	CTCCGACCTTCACCTTCC

IL-17A forward primer	TAGACTATGGAGAGCCGACC

IL-17A reverse primer	GGCAGAACTGATAATAGTGC

IL-17RAforward primer	AGGTCCAGCCCTTCTTCAGCA

IL-17RA reverse primer	GCTTGGGAACTGTGGTATTTGA

PI3K forward primer	GGGGATGATTTACGGCAAGATA

PI3K reverse primer	CACCACCTCAATAAGTCCCACA

AKT forward primer	TGAGAGAAGCCACGCTGTC
AKT reverse primer	CGGAGAACAAACTGGATGAA

pAKT forward primer	GCAGCACGTGTACGAGAAGA

pAKT reverse primer	GGTGTCAGTCTCCGACGTG

PTEN forward primer	AGACCATAACCCACCACAGC

PTEN reverse primer	ACACCAGTTCGTCCCTTTCC

Bcl-xL forward primer	AATGTCTCAGAGCAACCGGG

Bcl-xL reverse primer	AGTGGCTCCATTCACCGC

BAD forward primer	GGAATTCCATGTTCCAGATCCCAGA

BAD reverse primer	CTCGAGCTACTGGGAGGGGGCGG

**Table 4:** Twelve compounds identified in BSHXDS by HPLC Analysis.

NO	Compound	Quality	Retention time (min)
1.	Linoleic acid	280.2402	7.27
2.	Quercetin-3-O-*α*-L-rhamnopyranoside	448.1006	7.52
3.	Loganic acid 6′-O-*β*-D-glucoside	538.1898	7.57
4.	Kaempferol	286.0477	10.12
5.	Tanshinol B	296.1049	10.3
6.	2-(1-Methyl-2-hydroxyethyl)-3-hydroxy-8-methylphenanthrene-1,4-dione	296.1049	11.2
7.	Hydroxytanshinone II A	310.1205	11.6
8.	Isotanshinone III	296.1412	12.05
9.	Tanshinone II B	296.1412	12.87
10.	15,16-Dihydrotanshinone I	278.0943	13.12
11.	Diisobutyl phthalate	278.1518	13.29
12.	Tanshinone II A	294.1256	13.48

## Data Availability

The data used to support the findings of this study are available from the corresponding author upon request.

## Supplementary Materials

Supplementary Materials Appendix I: The chromatographic peaks of individual components of BSHXD in the positive and negative ion modes.
