# Supplementary material for: BuShen HuoXue Decoction Promotes Decidual Stromal Cell Proliferation via the PI3K/AKT Pathway in Unexplained Recurrent Spontaneous Abortion
Source: Evid Based Complement Alternat Med. 2020 Oct 5;2020:6868470. doi: 10.1155/2020/6868470 (PMC7556073; doi:10.1155/2020/6868470)
Supplement: Supplementary Materials — Appendix I: The chromatographic peaks of individual components of BSHXD in the positive and negative ion modes. [file 6868470.f1.pdf]

Appendix I

The chromatographic peaks of individual components of BSHXD in the positive and negative ion modes are as follows:

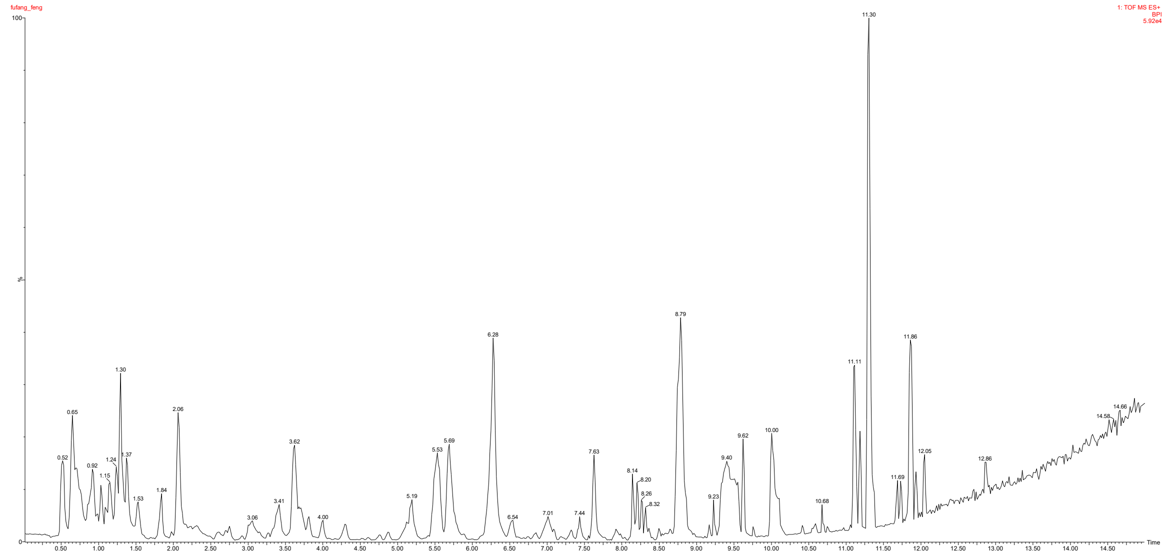

Positive ion chromatography of BSHXD

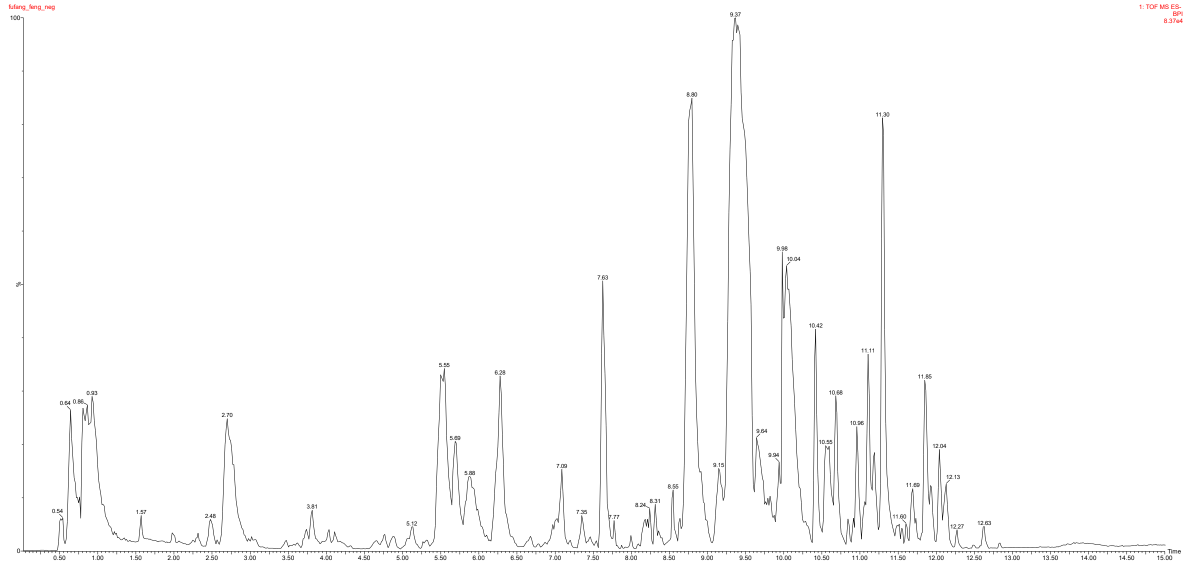

Negative ion chromatography of BSHXD

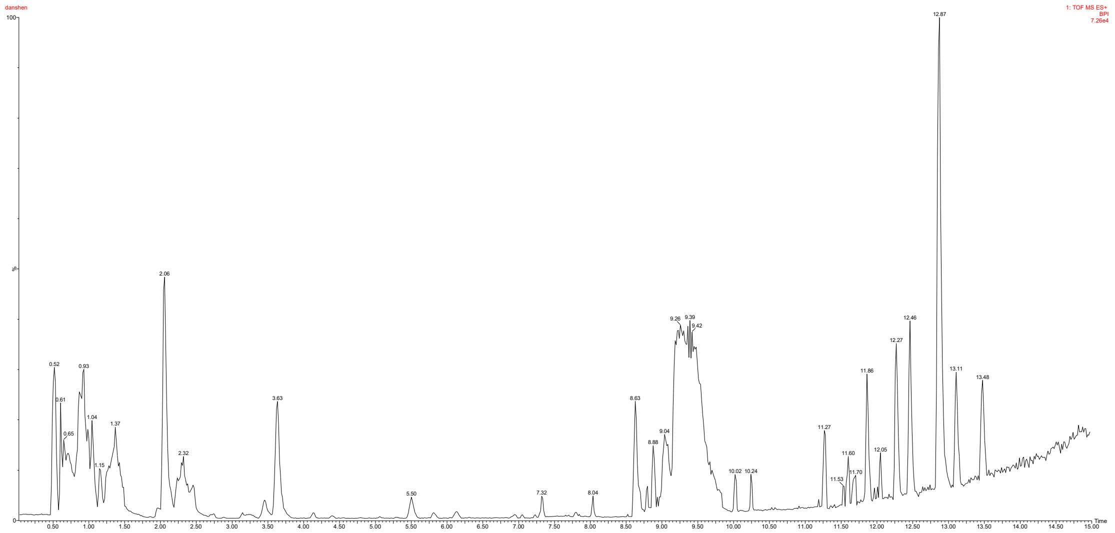

Positive ion chromatogram of Radix salviae miltiorrhizae

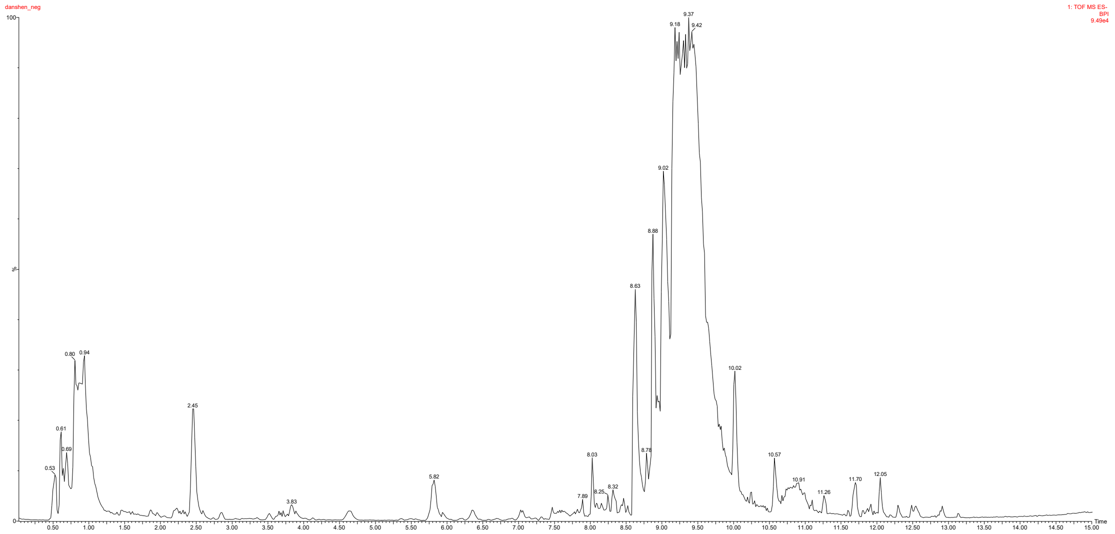

Negative ion chromatogram of Radix salviae miltiorrhizae

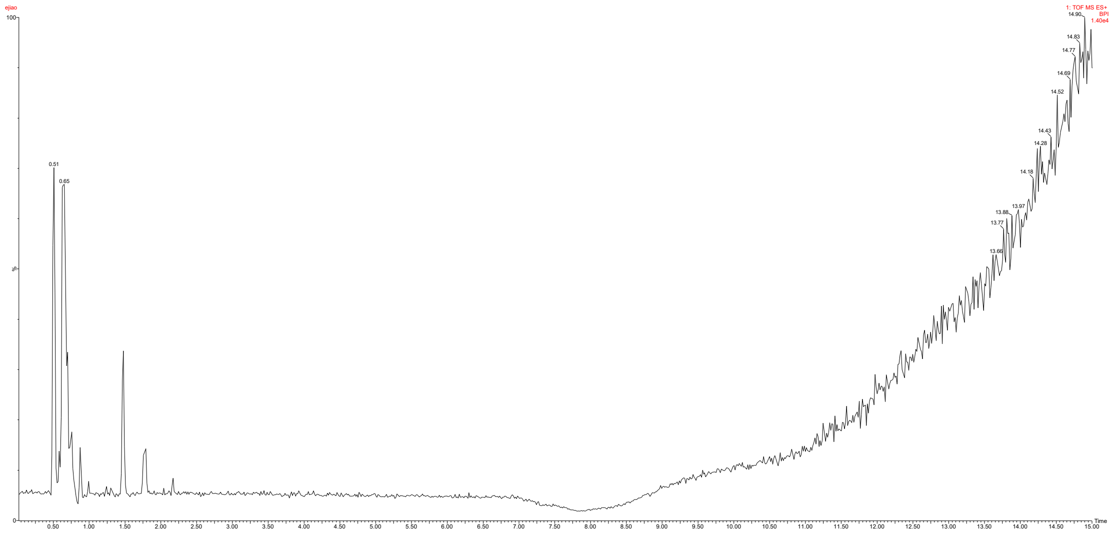

Positive ion chromatogram of Donkey-hide Glue

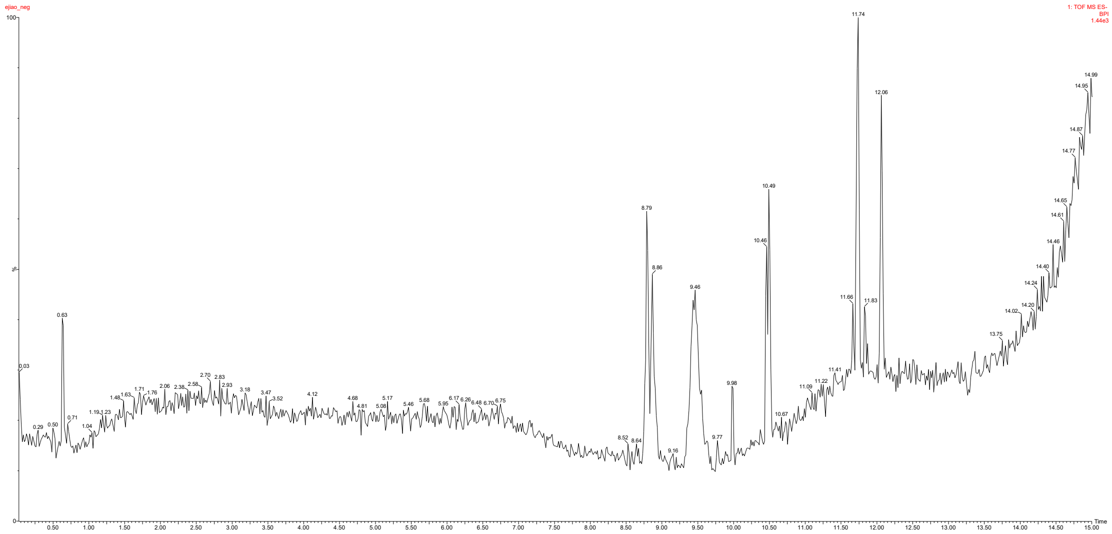

Negative ion chromatogram of Donkey-hide Glue

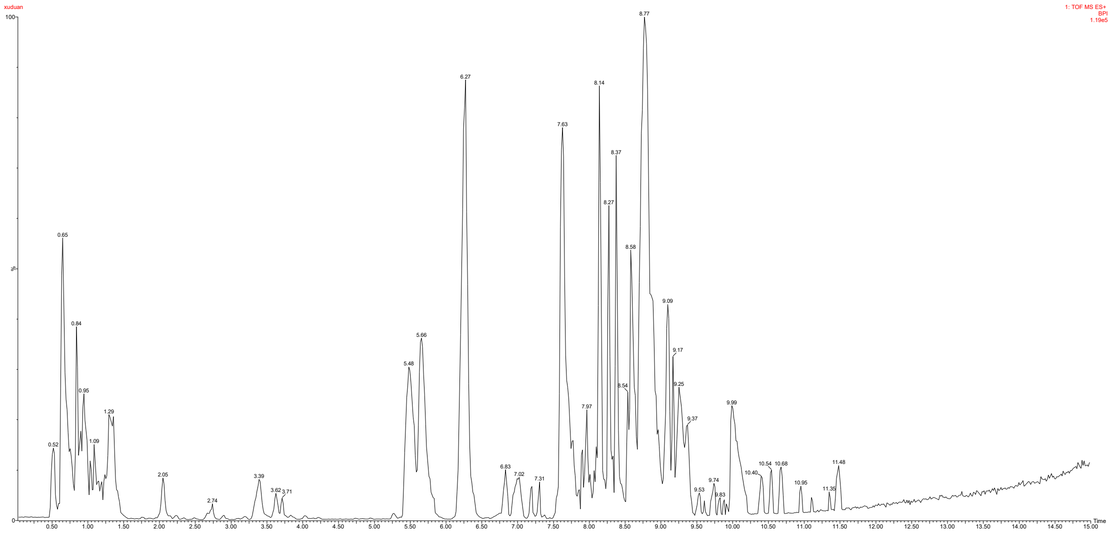

Positive ion chromatogram of Himalayan Teasel Root

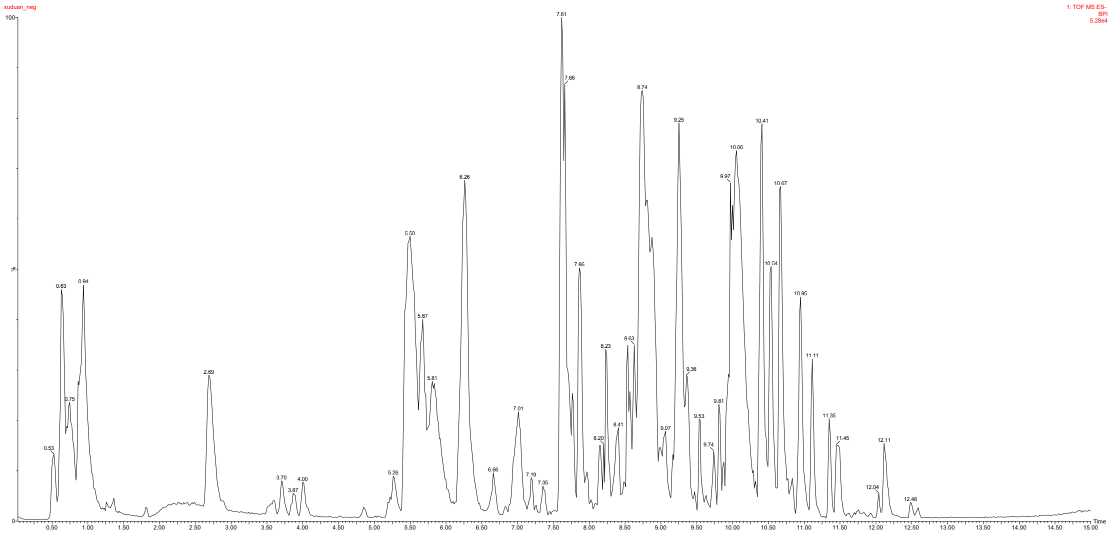

Negative ion chromatogram of Himalayan Teasel Root

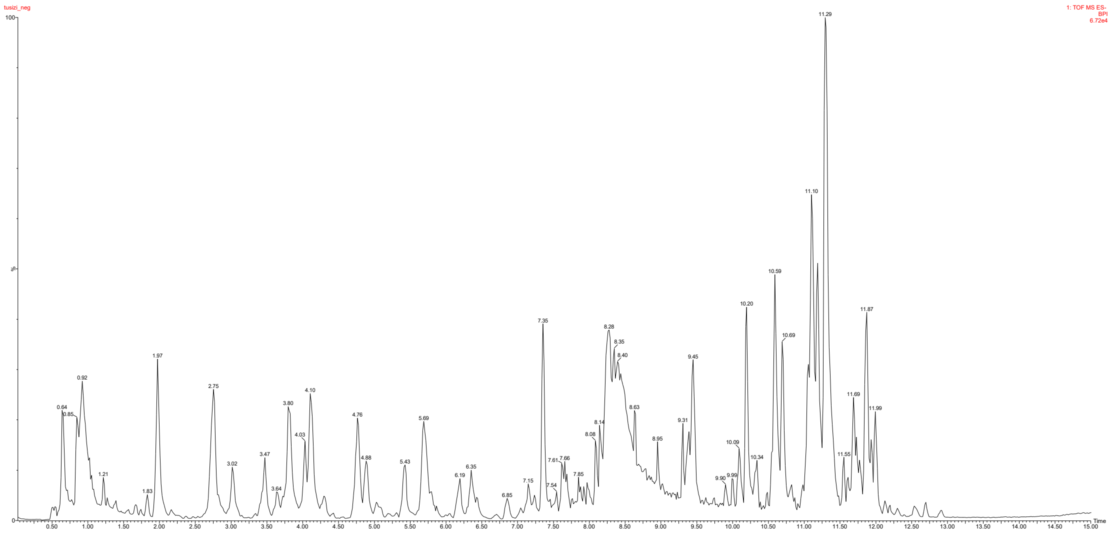

Positive ion chromatogram of Chinese Dodder Seed

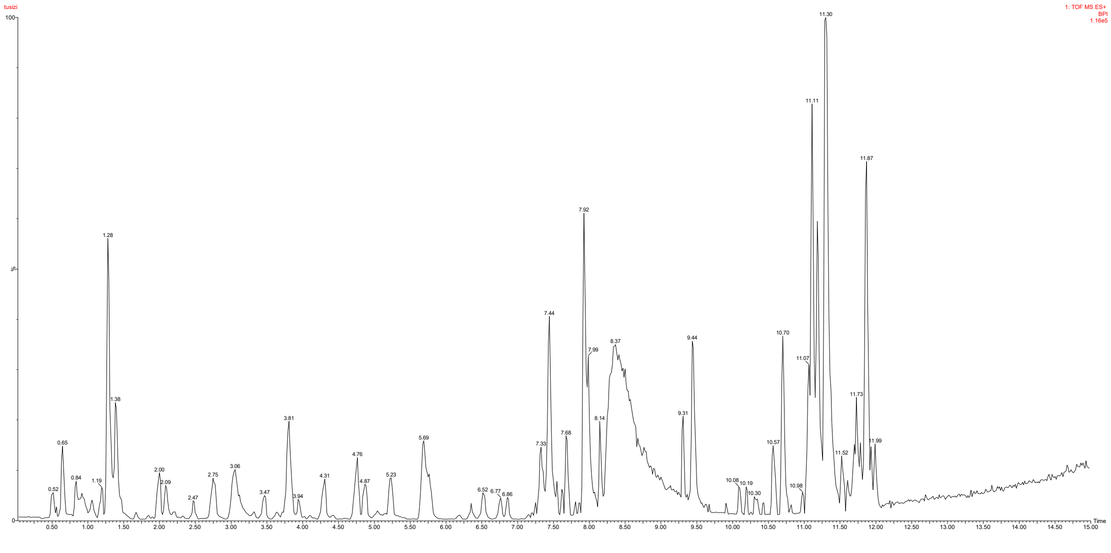

Negative ion chromatogram of Chinese Dodder Seed

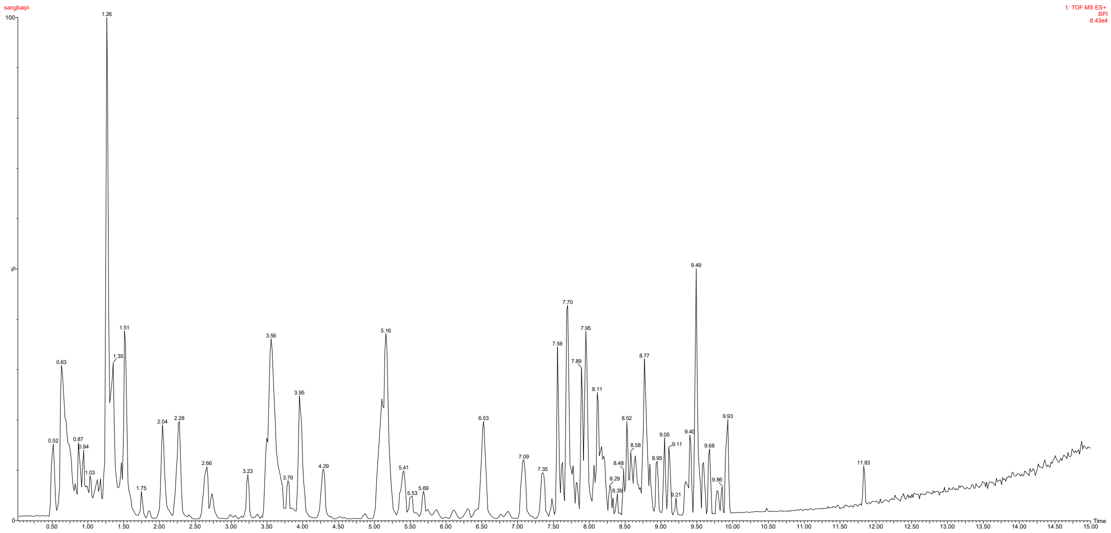

Positive ion chromatogram of Herba taxilli

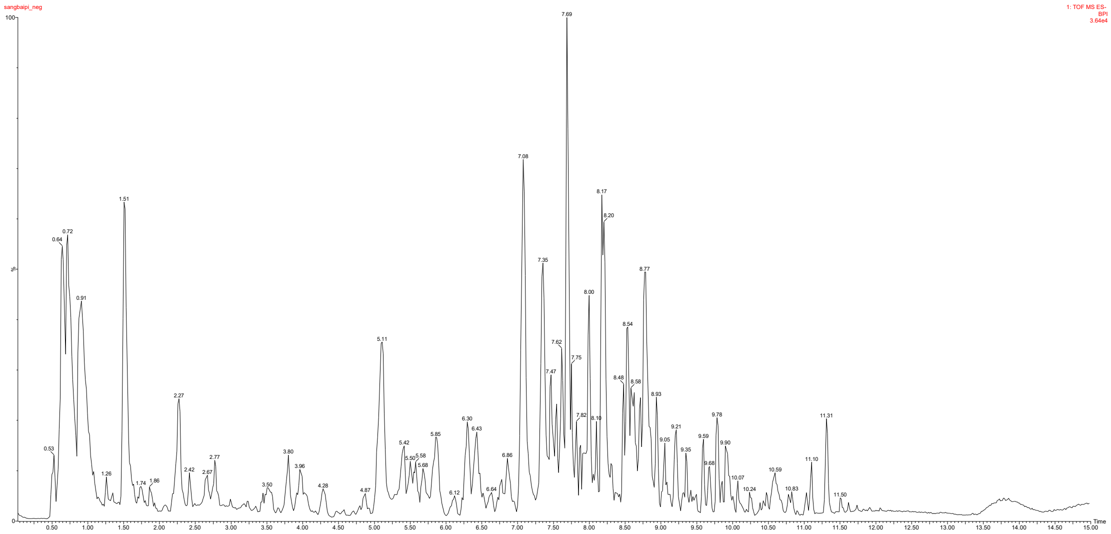

Negative ion chromatogram of Herba taxilli

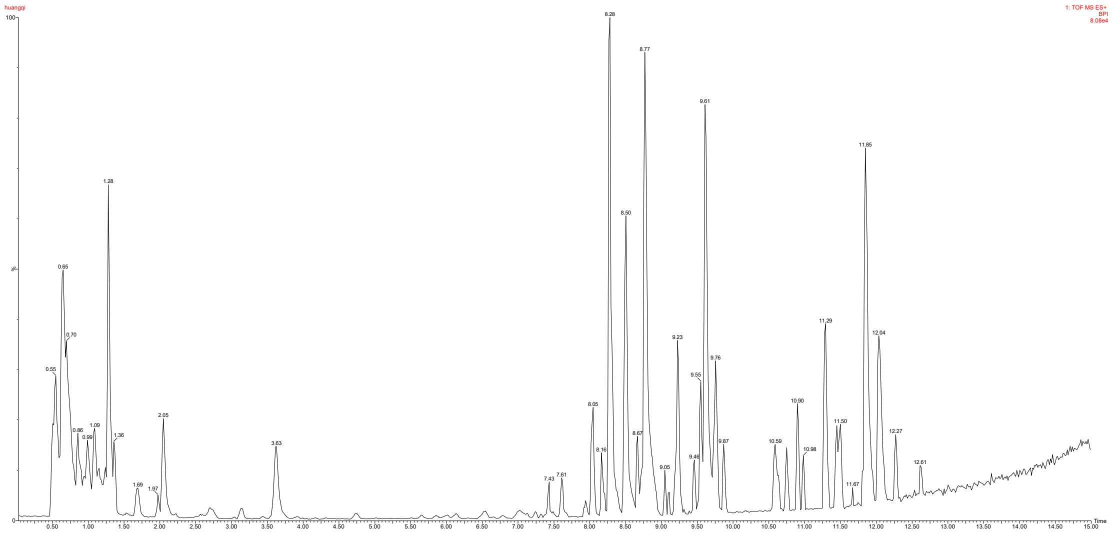

Positive ion chromatogram of Radix Astragali

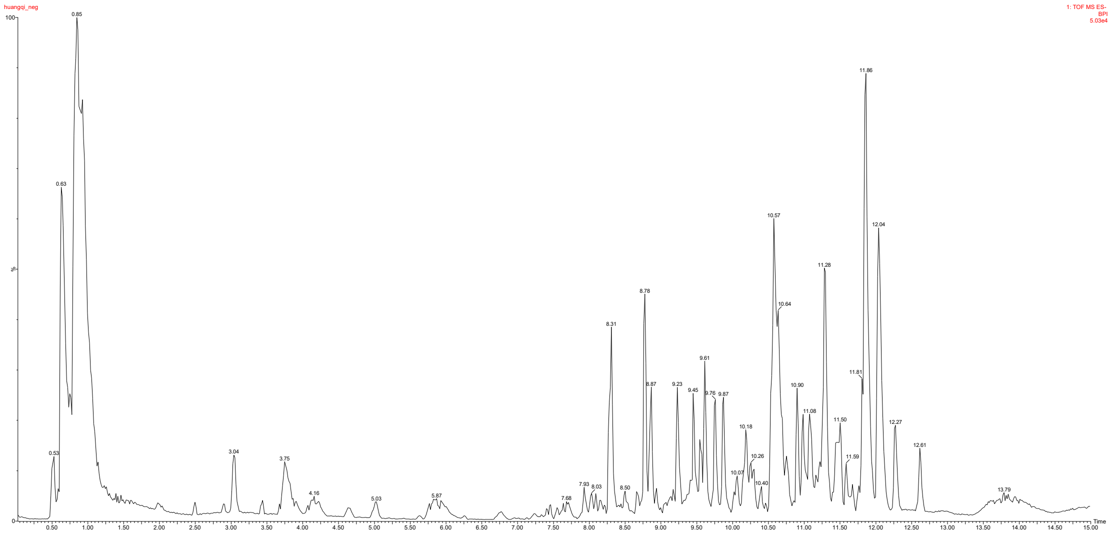

Negative ion chromatogram of Radix Astragali

Appendix II

Because the incomplete information of each traditional Chinese medicine included in the WATERS TCM database is not necessarily complete, so there will be some peaks without identification information. It can be seen from the chromatogram that the chromatographic peak of the whole formula is a collection of each single drug. In the experiment, positive and negative ions were collected separately, and most of the positive and negative ions responded. If positive and negative ions detected the same compound, only positive ion identification information was retained. We identified the components of the whole formula, and identified the source of the components in combination with the chromatographic peak of the single drug. A total of 75 compounds were identified. The results are as follows:

Radix salviae miltiorrhizae(21)

Mode	Compound name	mass number	Retention time (min)	Additive ion form
Positive ion mode	caffeic acid	180.0423	5.84	+H
	Lithospermic acid	538.1111	8.65	+H,+Na,+NH4
	Rosmarinic acid	360.0845	8.90	+H,+Na,+NH4
	salvianolic acid E	718.1534	8.99	+H,+Na,+NH4
	salvianolic acid B	718.1534	9.34	+H,+Na,+NH4
	Danshenxinkun A	296.1049	11.20	+H,+Na
	9-Hydroxy-1,6,6-trimethyl-6,7,8,9-tetrahydrophenanthro[1,2-b]furan-10,11-dione	310.1205	11.60	+H,+Na,+NH4
	(1R,10S)-10-hydroxy-1,6-dimethyl-10-(2-oxopropyl)-1,2-dihydronaphtho[1,2-g][1]benzofuran-11-one	296.1049	11.92	+H
	Isocryptotanshinone	296.1412	12.05	+H
	Dihydrotanshinone I	278.0943	12.28	+H,+Na
	1,6-dimethyl-1,2,8,9-tetrahydronaphtho[1,2-g][1]benzofuran-10,11-dione	280.1099	12.47	+H,+Na,-e
	Tanshinone IIB	296.1412	12.87	+H,+Na
	15,16-dihydrotanshinone	278.0943	13.12	+H,+Na,-e
	Tanshinone IIA	294.1256	13.48	+H,+Na,+NH4
Negative	2-(3,4-dihydroxyphenyl)-2-hydroxypropanoic acid	198.0528	2.49	-H,+HCOO

ion mode	3,4-Dihydroxybenzaldehyde	138.0317	3.86	-H
	Salvianolic acid A	494.1213	8.33	+HCOO
	Salvianolic acid C	492.1056	8.39	+HCOO
	(2R)-2-({(2E)-3-[2-(Carboxymethyl)-3,4-dihydroxyphenyl]-2-propenoyl}oxy)-3-(3,4-dihydroxyphenyl)propanoic acid	418.0900	8.72	-H
	methyl lithospermate	552.1268	9.02	-H
	6-Hydroxy-1,6-dimethyl-6,7,8,9-tetrahydrophenanthro[1,2-b]furan-10,11-dione	296.1049	10.30	+HCOO

Donkey-hide Glue（2）

mode	Compound name	mass number	Retention time (min)	Additive ion form
Positive ion mode	3,4-Dimethoxycinnamic acid	208.07356	0.51	-e,+Na
	Diisobutyl phthalate	278.15181	13.29	+H,+Na

Himalayan Teasel Root（3）

mode	Compound name	mass number	Retention time (min)	Additive ion form
Positive ion mode	Loganic acid	376.13695	6.27	+H,+Na,+NH4
	Loganic acid 6'-O-β-D-glucoside	538.18977	7.57	+H,+Na,+NH4
	asperosaponin VI	928.50317	10.02	+H,+NH4

Chinese Dodder Seed（18）

mode	Compound name	mass number	Retention time (min)	Additive ion form
Positive ion mode	N-methylcytisine	204.12626	3.74	+H
	d-Sesamin	354.11034	5.77	+H,+Na,+NH4
	Sophoranol	264.18378	5.85	+H
	Quercetin-3-galactose-7-glucoside	626.14830	6.92	+H,+Na
	New Cuscutoside B	796.24259	7.87	+H,+NH4
	Cuscutoside A	664.20034	8.24	+H,+Na,+NH4
	Quercetin-3-o-β-d-glucopyranoside	464.09548	8.34	+H,+Na
	Cuscutoside C	694.21090	8.48	+H

	Kaempferol-3-o-β-D-glucoside	448.10056	8.66	+H,+Na
	Cuscutoside B	664.20034	9.58	+H,+Na,+NH4
	Kaempferol	286.04774	10.12	+H,-e
	(1E,4E,8E)-α-humulene	204.18780	12.00	+Na
Negative ion mode	Sesamolin	370.10525	1.34	+HCOO
	New Cuscutoside A	826.25316	7.97	+HCOO
	Quercetin	302.04265	9.55	-H
	Palmitic acid	256.24023	9.69	+HCOO
	Octadecanoic acid	284.27153	10.60	+HCOO
	Matrine	248.18886	12.07	+HCOO

Herba taxilli（2）

Mode	Compound name	mass number	Retention time (min)	Additive ion form
Positive ion mode	dl-catechin	290.07904	0.72	+H
Negative ion mode	Quercetin-3-O-α-L-rhamnopyranoside	448.10056	7.52	-H

Radix Astragali(29)

Mode	Compound name	mass number	Retention time (min)	Additive ion form
	Polycanthine	261.13649	1.35	+Na
	Astragalosine A	209.06881	2.61	+NH4
	Linoleic acid	280.24023	7.27	+Na
	3'-methoxy-5'-hydroxyisoflavone-7-O-β-D-glucoside	446.12130	8.32	+H,+Na
	Formononetin	268.07356	8.36	+Na
	Calycosin-7-O-β-D-glucoside	446.12130	8.42	+H
	3,4-Dimethoxy-7,2’-O-diglucoside-7-O-glucoside	626.22107	8.71	+Na,+NH4,-e
	kaempferol-Methylether-3-7-O-β-D-glucopyranoside	462.11621	9.47	+H,+Na,+NH4
	Astragaloside V	946.51373	9.92	+H,+Na,+NH4
	Astragaloside VI	946.51373	10.60	+H,+Na,+NH4
	Astragalus membranaceus saponin B	652.41865	10.70	+H,+Na
Positive	Formononetin	268.07356	10.77	+H

ion mode	Isoastragaloside IV	784.46091	10.92	+H,+Na,+NH4
	Astragaloside III	784.46091	10.92	+H,+Na,+NH4
	Astragaloside IV	784.46091	11.00	+H,+Na,+NH4
	Isomucronulatol	302.11542	11.05	+H,-e
	Agroastragaloside III	1030.53486	11.09	+H,+Na
	Astragaloside II	826.47147	11.30	+H,+Na,+NH4
	Astragalus membranaceus Astragaloside II	622.40808	11.37	+H,+Na
	Agroastragaloside I	912.50825	11.60	+H,+Na,+NH4
	Agroastragaloside VIII	912.50825	11.77	+H,+Na,+NH4
	Isoastragaloside I	868.48204	12.28	+H,+Na,+NH4
	Astragaloside I	868.48204	12.28	+H,+Na,+NH4
	acetylastragaloside I	910.49260	12.63	+H,+Na,+NH4
Negative ion mode	Apigenin 7-glucoside	432.14203	3.06	+HCOO,-H
	methylnissolin	300.09977	5.06	-H
	Isoferulic acid	194.05791	5.96	+HCOO
	Kaempferide-4′-dimethyl ether-3 -glucoside	462.11621	9.47	+HCOO
	Agroastragaloside IV	988.52429	11.46	-H

Appendix III

The chromatographic peaks of the blood components in the blank group and the prescription for BXHXD in different time and in the mode of positive and negative ions are as follows:

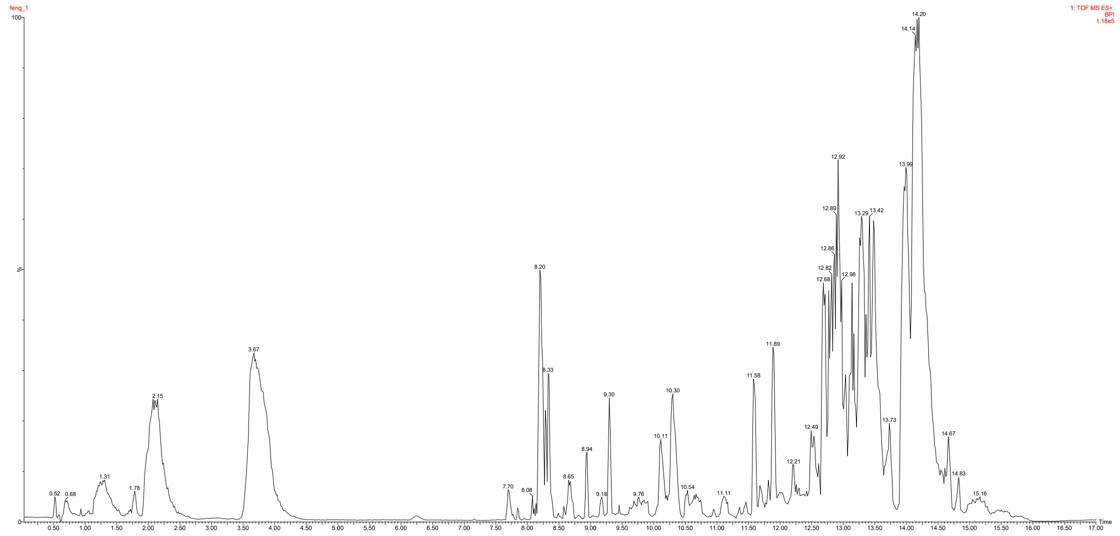

Chromatogram of blank serum in positive ion mode

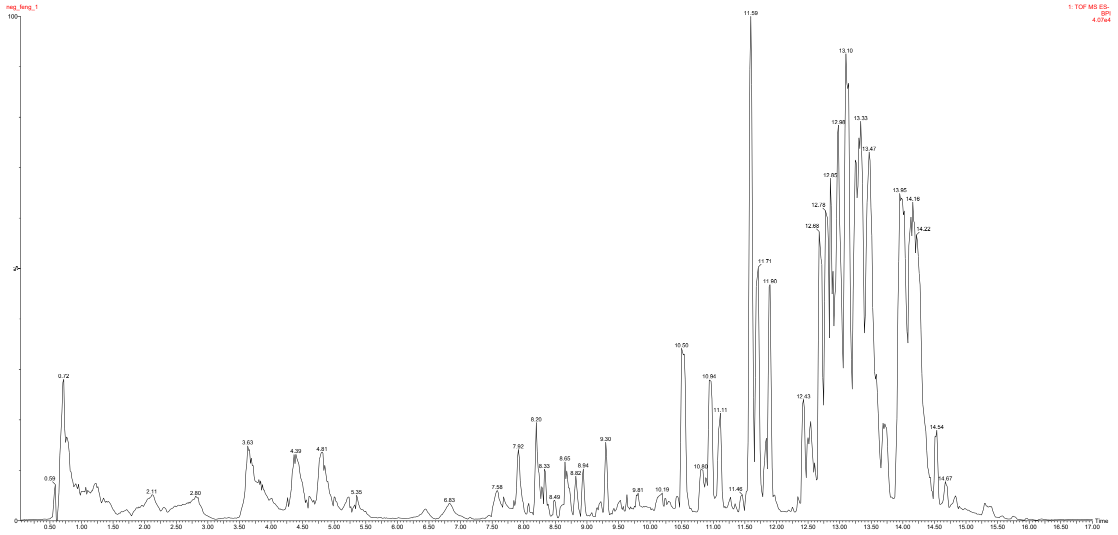

Chromatogram of blank serum in negative ion mode

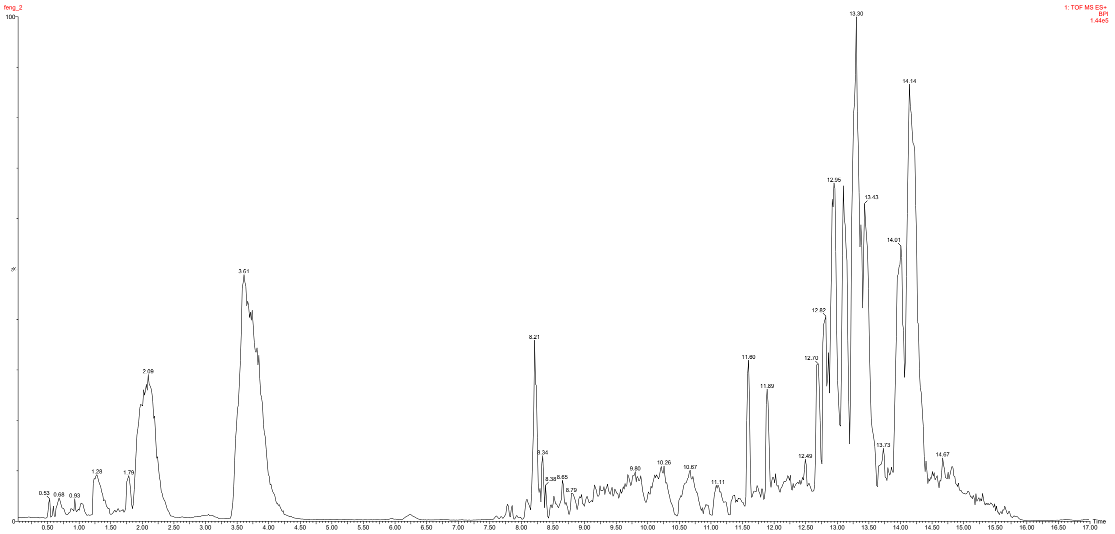

Chromatogram of BSHXDS in 15 min in positive ion mode

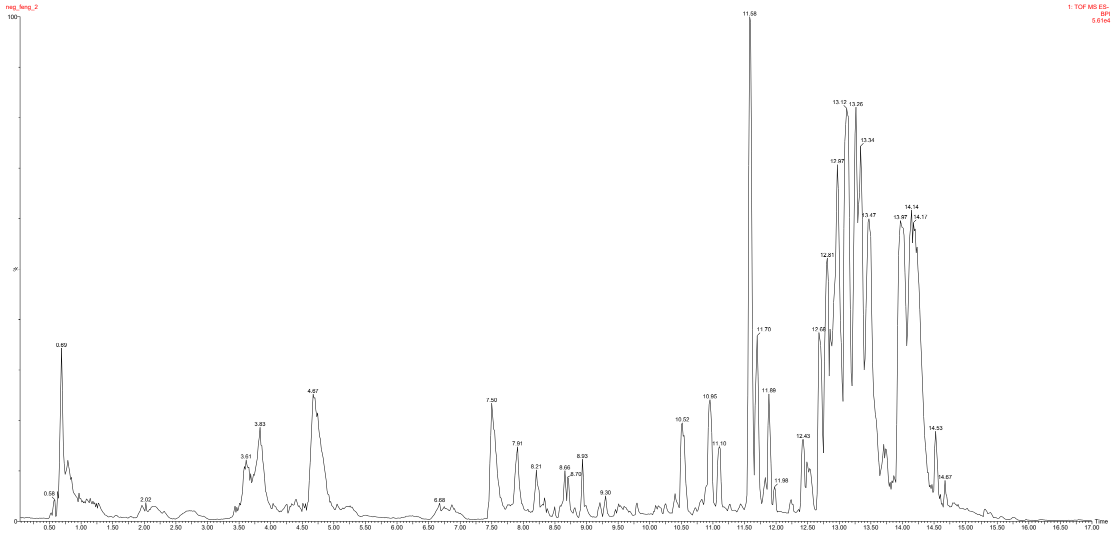

Chromatogram of BSHXDS for 15 min in negative ion mode

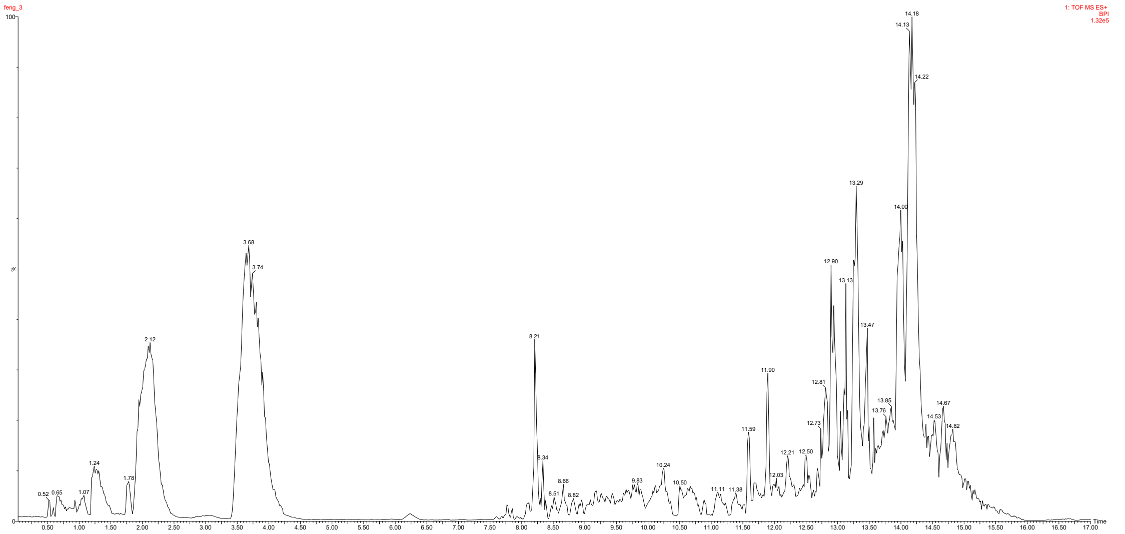

Chromatogram of BSHXDS in 30 min in positive ion mode

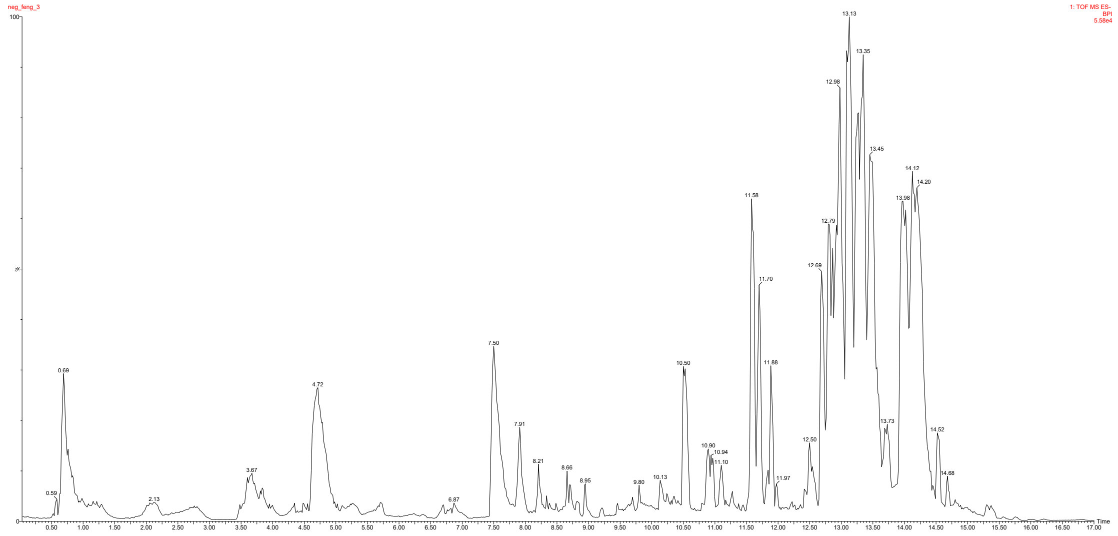

Chromatogram of BSHXDS for 30 min in negative ion mode

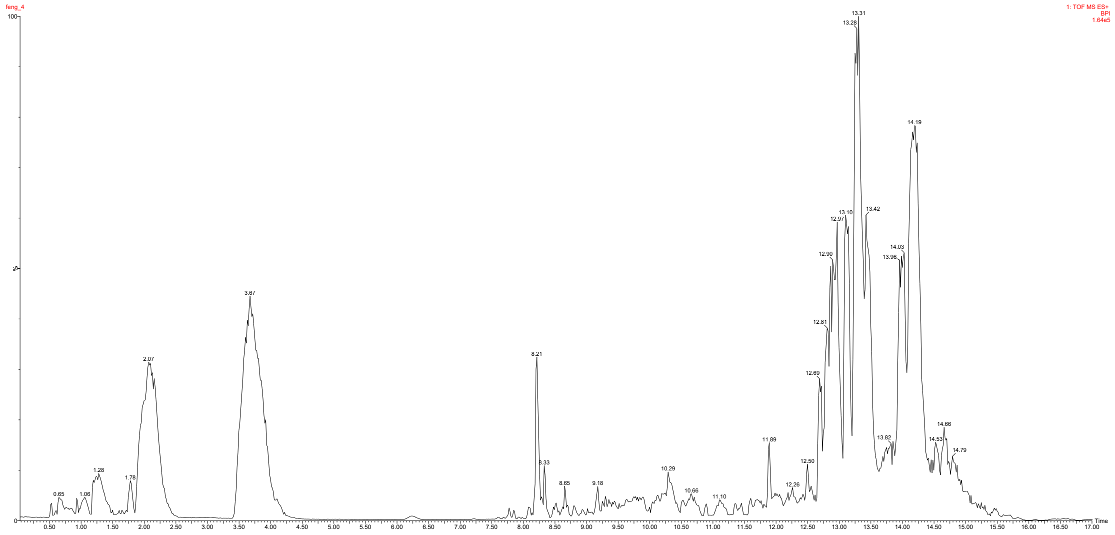

Chromatogram of BSHXDS in 1h in positive ion mode

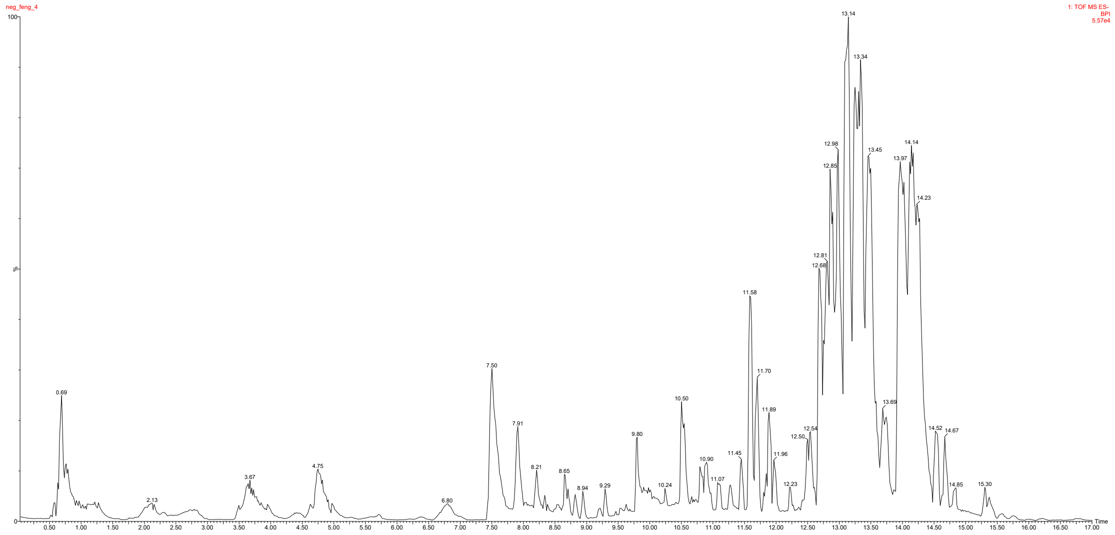

Chromatogram of BSHXDS for 1h in negative ion mode

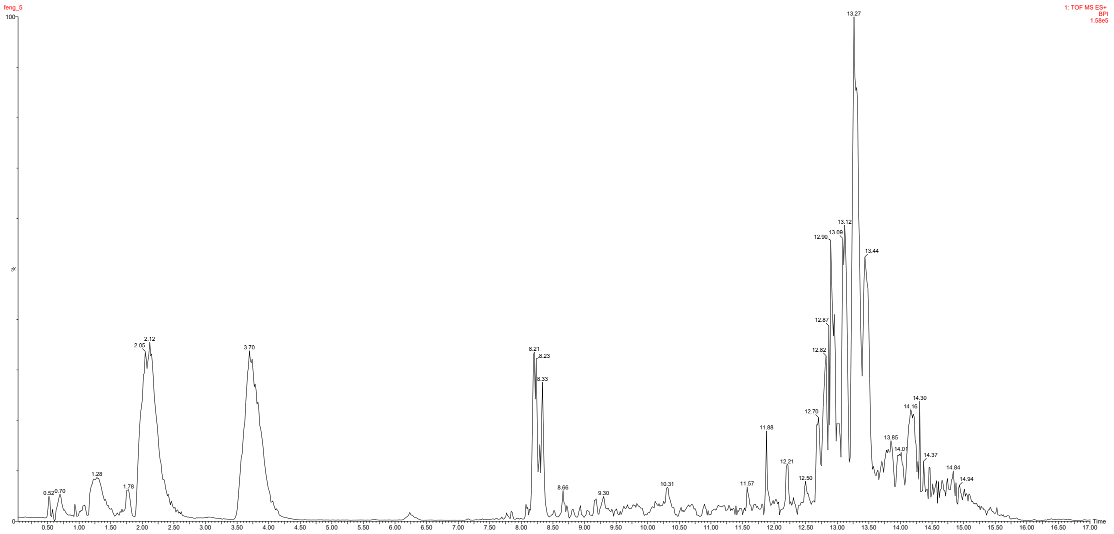

Chromatogram of BSHXDS in 2h in positive ion mode

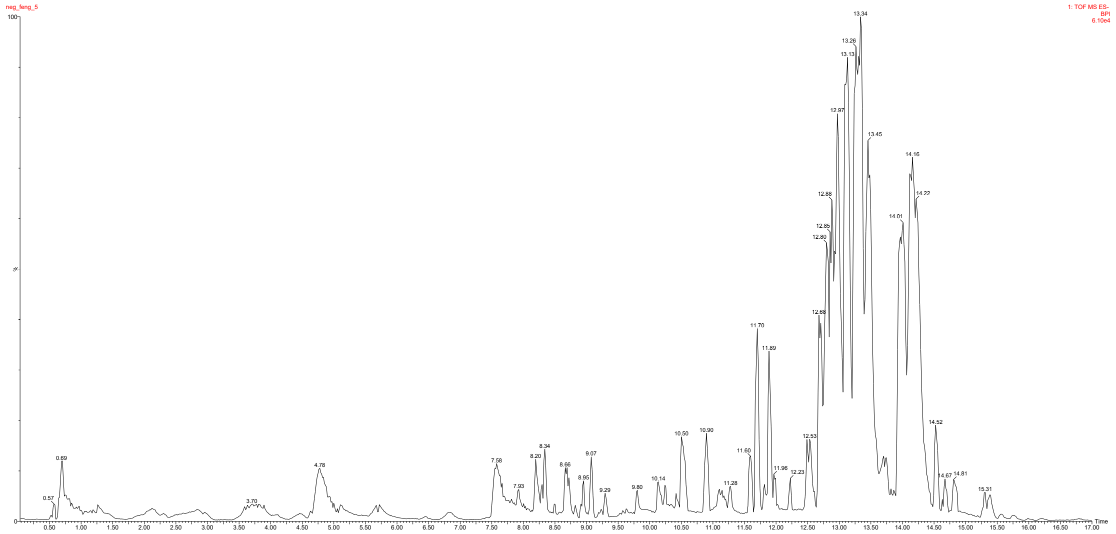

Chromatogram of BSHXDS for 2h in negative ion mode

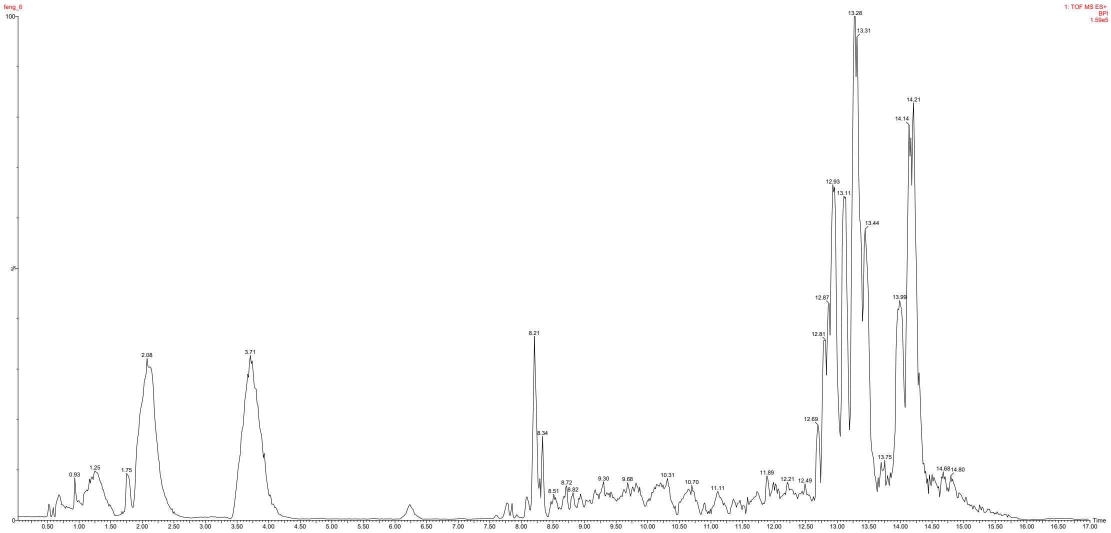

Chromatogram of BSHXDS in 4h in positive ion mode

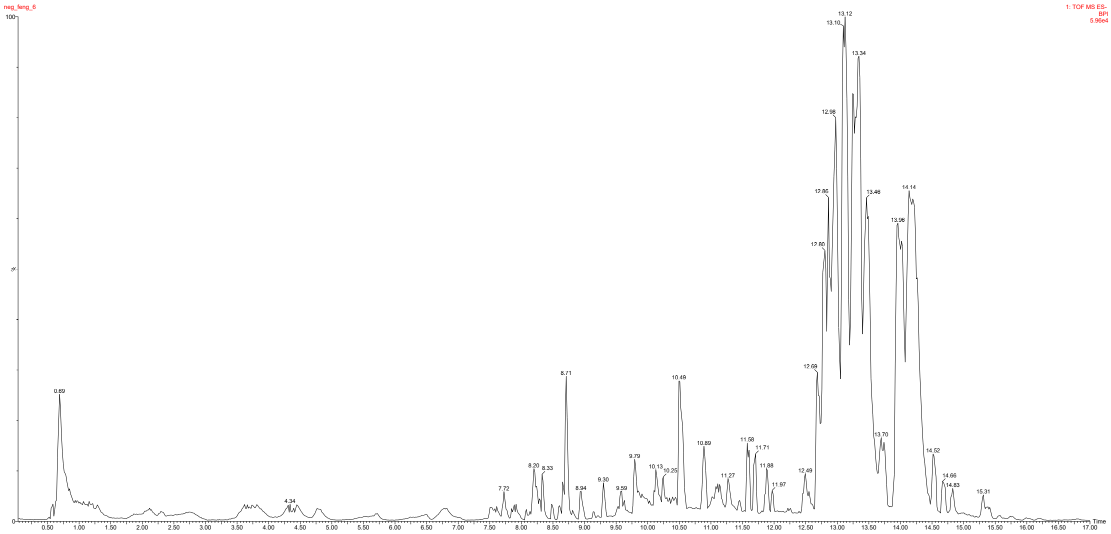

Chromatogram of BSHXDS for 4h in negative ion mode
